# Hypersensitivity reactions to iodinated contrast media: potential mechanisms and clinical management

**DOI:** 10.3389/fmed.2025.1582072

**Published:** 2025-05-07

**Authors:** Xia Zhong, Lihong Zhao

**Affiliations:** Department of Radiology, West China Hospital, Sichuan University, Chengdu, China

**Keywords:** iodinated contrast media, hypersensitivity reactions, epidemiology, mechanisms, clinical management

## Abstract

Iodinated contrast media (ICM) are indispensable in modern imaging, but hypersensitivity reactions (HSRs), ranging from mild urticaria to severe anaphylaxis, remain a significant and evolving clinical challenge. Although advancements in ICM formulations and HSR management, ongoing discussions and uncertainties persist, particularly regarding variable epidemiology, complex mechanisms, and debatable clinical management strategies. This review provides a comprehensive overview and insights into the epidemiology, clinical consequences, potential mechanisms, clinical management, and current controversies associated with HSRs to ICM. Ongoing research is critical, focusing on areas such as monitoring epidemiological trends, uncovering underlying mechanisms, improving risk prediction, and refining preventive, diagnostic, and therapeutic strategies. Overall, as the use of ICM continues to rise, balancing their diagnostic benefits with effective management of HSRs is essential to optimizing patient safety and clinical outcomes.

## 1 Introduction

Iodinated contrast media (ICM) remain indispensable in modern diagnostic imaging, particularly in computed tomography (CT) and angiography, where they critically enhance the visualization of vascular pathologies, neoplastic lesions, and inflammatory processes (1). While contemporary formulations prioritize safety and efficacy through osmolarity optimization ranging from low-osmolar monomers (e.g., iopamidol, iohexol) to iso-osmolar dimers (e.g., iodixanol) and chemical innovation, their clinical utility continues to be challenged by unpredictable hypersensitivity reactions (HSRs) (2–4). Recent advancements such as biodegradable iodinated polydisulfides and nanoparticle-based media demonstrate progress toward reducing adverse reactions and improving imaging resolution (5–7). However, HSRs, spanning mild urticaria to fatal anaphylaxis that can occur in rare cases, persist as a critical safety concern (8–10). This challenge assumes greater urgency because of the escalating global reliance on contrast-enhanced imaging and persistent inconsistencies in HSR reporting frameworks.

Hypersensitivity reactions are temporally stratified into immediate and non-immediate subtypes (11, 12). Immediate reactions typically occur within an hour following ICM administration, while non-immediate reactions occur hours to days after ICM exposure. While intradermal skin testing has emerged as a tool to identify cross-reactive ICM and guide alternative ICM selection, its predictive value remains debated (13, 14). Similarly, premedication protocols, such as corticosteroids and antihistamines, also lack standardized guidelines, particularly for high-risk populations with prior reactions or comorbidities (15, 16). Current studies disproportionately emphasize epidemiological patterns or clinical management algorithms, leaving mechanistic insights, risk assessment, and interdisciplinary syntheses underdeveloped.

As the use of ICM continues to increase, so does the need for heightened vigilance regarding possible HSRs. This review comprehensively summarizes the advances in epidemiology, potential mechanisms, clinical consequences and complications, and clinical management of HSRs to ICM. The findings and insights are intended to inform clinical decision-making and improve patient safety during imaging procedures involving ICM. Meanwhile, this review addresses ongoing controversies regarding HSRs to ICM and outlines key focus for future research, emphasizing the need to refine preventive, diagnostic, and therapeutic strategies, particularly in high-risk populations.

## 2 Epidemiology of HSRs to ICM

The incidence of HSRs to ICM varies significantly across studies (17, 18). A large-scale multicenter study involving 196,081 patients reported an overall HSR prevalence of 0.73%, with severe reactions occurring in 0.01% of cases (19). Specific analyses for different ICM reveal noticeable differences (Table 1). For example, ioversol, an often used low-osmolar and non-ionic ICM, demonstrates an incidence range of 0.20–0.66% in adults (20), while high-osmolar ionic ICM like diatrizoate show rates up to 7% (21). Immediate reactions dominate clinical presentations, accounting for 67% of HSR cases in Italian cohorts (15), with cutaneous manifestations (urticaria, pruritus) occurring in 0.12–1.15% of exposures (22). A Korean study reported that immediate HSRs were more common than non-immediate reactions, with an overall occurrence rate of 0.37% for adverse drug reactions related to ICM (23). Though less frequently recognized, non-immediate reactions manifest as various cutaneous symptoms and are also documented but may be under recognized (12). Severe HSRs to ICM were relatively rare, and previous investigations reported it occurring in 1.92% of HSR cases, while mild reactions were up to 86.2% (24, 25).

**Table 1:** Characteristics and prevalence of HSRs across different ICM.

ICM name	Structural and chemical properties			HSR rate (%)
	Ionic/non-ionic	Monomer/dimer	Osmolarity	
Iobitridol	Non-ionic	Monomeric	Low-osmolar	0–3.6 (37)
Iohexol	Non-ionic	Monomeric	Low-osmolar	0–1.6 (19, 94)
Iomeprol	Non-ionic	Monomeric	Low-osmolar	0–1.6 (19, 94)
Iopamidol	Non-ionic	Monomeric	Low-osmolar	0–2.3 (19, 94)
Iopromide	Non-ionic	Monomeric	Low-osmolar	0–1.0 (94)
Ioversol	Non-ionic	Monomeric	Low-osmolar	0–1.0
Ioxilan	Non-ionic	Monomeric	Low-osmolar	0–1.4 (22)
Iopentol	Non-ionic	Monomeric	Low-osmolar	0–1.7 (95)
Iodixanol	Non-ionic	Dimeric	Iso-osmolar	0–2.1 (37)
Iotrolan	Non-ionic	Dimeric	Iso-osmolar	0–16.4 (96)
Ioxaglate	Ionic	Dimeric	Low-osmolar	0–9.5 (97)
Iothalamate	Ionic	Monomeric	High-osmolar	0–5.1 (21)
Ioxithalamate	Ionic	Monomeric	High-osmolar	Uncertain
Diatrizoate	Ionic	Monomeric	High-osmolar	0–7.0 (21)
Metrizoate	Ionic	Monomeric	High-osmolar	0–9.5 (98)
Iopanoic acid	Ionic	Monomeric	High-osmolar	Uncertain
Iotroxic acid	Ionic	Monomeric	High-osmolar	Uncertain

Although the overall prevalence of HSRs to ICM has remained relatively stable over the past decade, it varies across studies due to differences in populations, study design, definitions of HSRs, types of ICM used, premedication protocols, and geographical or institutional factors. An Italy study indicated demographic disparities, with females exhibiting 1.5-fold higher susceptibility than males (15). Selection and reporting bias also influence the prevalence, with non-representative populations and underreporting or overreporting HSRs skewing results (26, 27). Retrospective studies face inherent limitations in data accuracy, particularly regarding non-immediate reactions that may be misattributed to concurrent medications (15, 28). Large sample sizes, as seen in multicenter studies, tend to provide more reliable prevalence estimates by reducing error margins and increasing statistical power (19, 24). Discrepancies in defining and classifying HSRs contribute to variations, with some studies including only severe reactions and others considering mild to severe cases (29, 30). Limited access to alternative imaging modalities can increase HSR incidence due to greater reliance on ICM (25). The introduction of new ICM has influenced HSR incidence, with some newer ICM demonstrating lower rates than traditional ICM (24). Geographical or institutional factors also influence reported rates. For example, Korean pharmacovigilance data document lower HSR incidence (0.37%) compared to European cohorts (0.73%), reflecting potential differences in ICM utilization patterns or genetic susceptibility (19, 23). Despite the low global incidence of HSRs to ICM, future research needs to adopt standardized methodologies and definitions to ensure consistency and comparability of findings across different studies.

## 3 Clinical consequences and complications of HSRs to ICM

Hypersensitivity reactions to iodinated contrast media pose a significant concern in medical imaging, with potential clinical consequences ranging from mild symptoms to life-threatening complications. These reactions can be immediate or non-immediate, affecting patient safety and complicating diagnostic and therapeutic procedures. Immediate HSRs may present as mild symptoms, such as urticaria, pruritus, and localized facial edema, or more severe manifestations, like anaphylaxis, which can be life-threatening (13, 31). Non-immediate HSRs are often characterized by skin reactions, such as maculopapular exanthems, and can lead to severe conditions, including Stevens-Johnson syndrome or toxic epidermal necrolysis, in some cases (12). Although the incidence of non-immediate HSRs is generally lower than that of immediate reactions, they remain a significant risk, particularly in patients with a history of drug allergies or prior HSRs to ICM (32, 33). In patients undergoing repeated imaging, these reactions may be misattributed to other medications or conditions, delaying appropriate treatment (12, 14, 34). Moreover, severe HSRs to ICM are of particular concern due to their potential to cause significant morbidity, often requiring immediate medical intervention (35).

HSRs to ICM also affect patients’ psychological well-being and prognosis, imposing a substantial economic burden on healthcare systems. Patients who experience these reactions may develop anxiety or fear of future imaging procedures involving ICM, which can lead to avoidance of essential diagnostic tests. This avoidance may delay diagnosis and treatment, adversely impacting prognosis. Studies show that patients with a history of HSRs to ICM often experience increased anxiety and stress, which may exacerbate their health conditions and complicate medical management (15, 29). Additionally, the need for alternative imaging modalities, such as gadolinium-based ICM, can complicate patient management due to potential contraindications or reduced efficacy (33). The recurrence of HSRs upon re-exposure requires careful planning, including premedication, which adds complexity to clinical workflows and increases the burden on healthcare providers (17, 35).

Economically, HSRs to ICM contribute to increased healthcare burden, involving direct expenses and indirect costs. Direct expenses arise from the need for alternative imaging, additional diagnostic tests, premedication, extended hospital stays, and emergency interventions for severe reactions (33, 36). Although premedication has been shown to reduce the incidence of HSRs, its effectiveness varies, and breakthrough reactions may still occur, necessitating further medical attention (17). Meanwhile, skin testing and other diagnostic evaluations to identify patients at risk of HSRs contribute to the healthcare burden (13). Furthermore, the need for close monitoring and potential treatment during imaging procedures strains healthcare staff and facilities, particularly in high-volume imaging centers (37). Emergency interventions in severe HSRs demand immediate access to emergency care facilities and trained personnel, emphasizing the importance of preparedness and adequate resource allocation in healthcare settings (36). Indirect costs of HSRs to ICM include compromised patient well-being, productivity losses due to delayed recovery, and increased healthcare utilization (19, 24). Understanding the mechanisms, risk stratification, and management strategies for HSRs to ICM is essential to mitigate these costs.

## 4 Potential mechanisms of HSRs to ICM

### 4.1 Immune and non-immune responses

HSRs to ICM can be categorized into immune-mediated and non-immune-mediated mechanisms. Immune-mediated HSRs are typically classified as either immunoglobulin E (IgE)-mediated or non-IgE-mediated (immune responses that do not involve IgE antibodies). Immediate HSRs, also known as type I reactions, are primarily IgE-mediated. Upon re-exposure to the allergen, IgE antibodies bound to mast cells and basophils trigger the release of histamine and other inflammatory mediators, leading to symptoms such as urticaria, angioedema, and anaphylaxis (38–40). Skin testing can help diagnose IgE-mediated reactions, with positive skin tests confirming immediate HSRs to ICM (39, 41). Non-IgE-mediated immune reactions may involve complement activation, activating basophils and mast cells through alternative pathways (38, 42). On the other hand, non-immediate HSRs are mainly T-cell mediated, and the lymphocyte transformation test (LTT) has been used as an in-vitro diagnostic tool, although its sensitivity varies. Recent advancements have improved the diagnostic sensitivity of LTT by incorporating autologous monocyte-derived dendritic cells, which serve as professional antigen-presenting cells and promote T-cell activation and cytokine production (43–45).

Non-immune-mediated HSRs, known as pseudo-allergic reactions, do not involve traditional immune pathways but result from direct activation of mast cells, complement system activation, and membrane effects. The Mas-related G protein-coupled receptor X2 (MRGPRX2), expressed on mast cells, mediates non-IgE-dependent mast cell degranulation in response to ICM like iopamidol and iohexol, triggering the release of inflammatory mediators such as histamine and tumor necrosis factor-alpha (TNF-*α*) (4, 46, 47). Recent studies found that elevated MRGPRX2 levels are associated with an increased risk of severe reactions, and MRGPRX2 may be a biomarker for predicting ICM-induced anaphylaxis (46). Meanwhile, ICM can directly activate the complement system, producing anaphylatoxins like C3a and C5a, which stimulate mast cells and basophils, contributing to the inflammatory response observed in some patients following ICM administration (38). Moreover, ICM can interact with cell membranes, activating intracellular signaling pathways that lead to the release of inflammatory mediators (38). Overall, the heterogeneity of mechanisms underlying HSRs to ICM is complex, involving both immune and non-immune pathways. Individual patients may experience HSRs to ICM through a combination of diverse pathways (38), and understanding these interactions is crucial for improving diagnosis and management.

### 4.2 Chemical characteristics of ICM and HSRs

The chemical characteristics of ICM, including iodine atoms, aromatic rings, side-chain chemistry, ionicity, osmolality, dimer/monomer structure, hydrophilicity/lipophilicity, and surface charge, play a crucial role in their immunogenicity and the pathogenesis of HSRs. The specific chemical groups, such as iodine atoms and benzene rings, can act as haptens, binding to proteins and forming hapten-protein complexes that the immune system recognizes as foreign, thus triggering an immune response in sensitized individuals (9). This process involves the activation of mast cells and basophils, releasing histamine and other mediators that cause the symptoms of HSRs (9, 17). ICM with reactive side chains (e.g., iohexol’s triiodinated benzene ring) may act as haptens, covalently binding to host proteins such as albumin to form immunogenic complexes, which trigger IgE production in rare cases, leading to immediate HSRs (24, 48). Patients with prior IgE sensitization to iopamidol show positive skin tests, supporting this mechanism (48). Non-ionic ICM such as iodixanol with N-(2,3-dihydroxypropyl) carbamoyl side chains generate metabolites that bind to MHC class II molecules, activating CD4 + T cells and release IL-5 and IFN-*γ*. This underlies maculopapular rashes or drug eruptions seen 24–72 h post-exposure and also supports the higher non-immediate HSRs to odixanol compared to ioversol (34, 49). Ionic high-osmolality ICM such as diatrizoate can induce osmotic stress, directly activating mast cells via TRPV1/TRPA1 channels and calcium influx, leading to histamine and tryptase release, as well as higher rates of immediate HSRs (31, 37). Non-ionic low-osmolality ICM such as iohexol and iopamidol lead to reduced osmotic stress but retain risk via other pathways such as T-cell activation (24, 34).

Meanwhile, dimeric ICM, such as iodixanol, prolongs tissue retention due to its larger molecular size, increasing immune exposure and non-immediate HSR risk (37, 49). Monomeric ICM, like iomeprol, possess smaller sizes and can reduce immune recognition but may enhance direct mast cell effects (31). Hydrophilic side chains (e.g., ioversol’s hydroxyl groups) are associated with lower HSR risk due to reduced protein binding and complement activation (24). Lipophilic ICM such as iopromide penetrates cell membranes more readily, increasing the production of reactive oxygen species (ROS), which activate MAPK/NF-κB pathways and upregulate pro-inflammatory cytokines like TNF-*α* and IL-6 (31, 37). ICM surface charge (e.g., negatively charged ionic ICM) also activates the alternative complement pathway, generating C3a/C5a anaphylatoxins, further amplifying mast cell activation (17, 37). Molecular dynamics simulations have shown that the entry behavior of ICM molecules into the membrane depends on their chemical structure, affecting the alignment of phospholipid headgroups and the order parameters of phospholipid tails. This interaction can lead to membrane thickness fluctuations, which may trigger immune responses (50). The structural similarity of ICM to thyroid hormones has also raised concerns about their potential to interact with hormone receptors involved in endocrine regulation, potentially acting as endocrine-disrupting chemicals (51). This interaction could contribute to the HSRs observed with ICM, but more research is needed to explore these mechanisms.

Moreover, cross-reactivity among different ICM is a crucial consideration in managing HSRs, as patients allergic to one ICM may also react to others, highlighting the importance of understanding the three-dimensional structure of ICM and their interaction with immune cells when selecting safe alternatives (29, 52). According to these mechanisms, switching ICM to those with distinct side-chain profiles, such as iopamidol to iohexol, reduces the recurrence risk of HSRs by 67% (24, 30). Meanwhile, antihistamines and glucocorticoids mitigate non-immune pathways but are ineffective for IgE/T cell-mediated reactions (19, 37). However, some unresolved issues need to be further explored. For example, the exact epitopes of ICM recognized by T cells or IgE remain unidentified, and mechanisms underlying geographic variability in HSR profiles (e.g., higher iopromide-linked angioedema in Asia vs. the US) are unclear (31).

### 4.3 Patient-specific mechanisms

**Figure 1:**
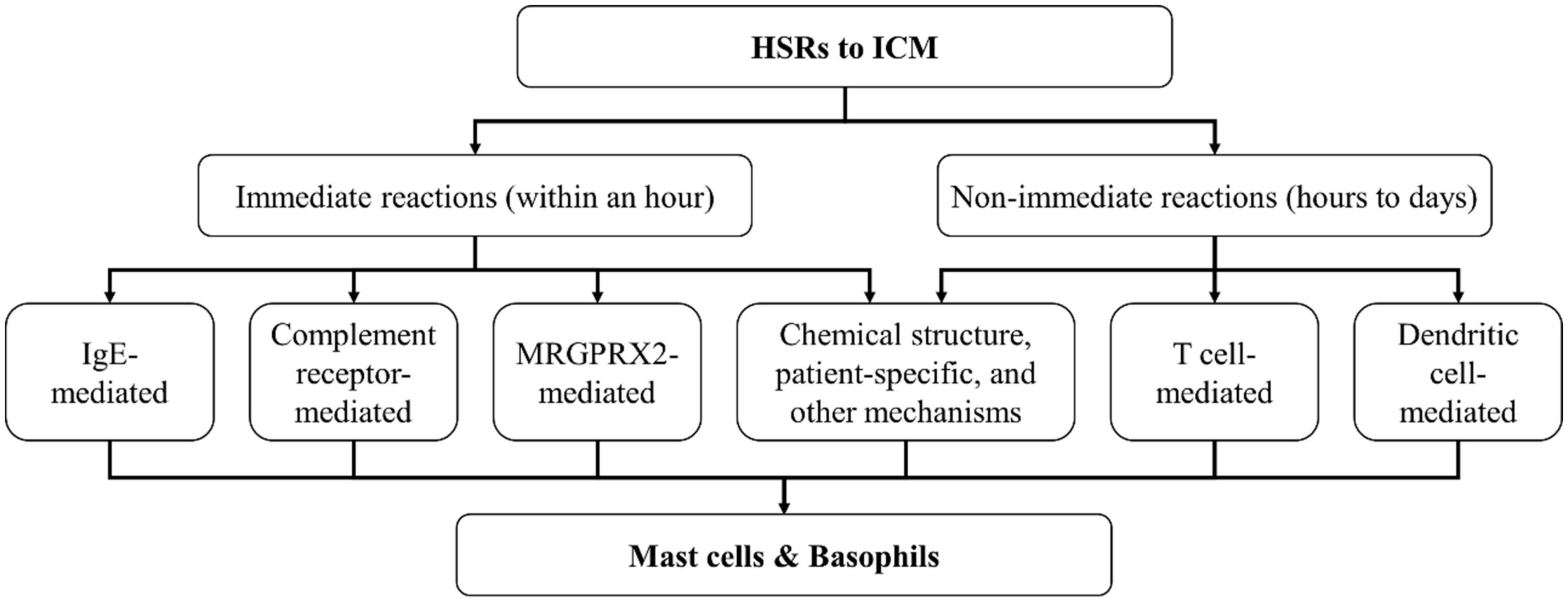
Potential mechanisms of HSRs to ICM. HSRs, hypersensitivity reactions; ICM, iodinated contrast media; IgE, immunoglobulin E; MRGPRX2, Mas-related G protein-coupled receptor X2.

Patient-specific factors, including genetics, age, gender, and underlying health conditions, further complicate the heterogeneity of HSRs to ICM. Genetic factors may play a role, as genome-wide association studies have suggested certain loci are associated with skin reactions to non-ionic ICM, though further research is needed to fully understand these links (53). Genetic predisposition, such as specific human leukocyte antigen (HLA) alleles, has been linked to ICM-induced HSRs, and HLA-B*38:02 is associated with an increased risk of immediate HSRs to iopromide, suggesting the potential for genetic screening to predict individual risk (54). Age and gender also contribute to the susceptibility of HSRs to ICM, possibly due to age-related immune function changes and hormonal differences (15). Other underlying conditions, such as a history of allergies, comorbidities like diabetes and cardiovascular diseases, and oncological status, can modulate immune responses, thereby participating in the pathogenesis of HSRs to ICM (30, 55). These patient-specific mechanisms emphasize the need for personalized prevention and management strategies for HSRs to ICM. In addition, some unknown mechanisms regarding HSRs to ICM still need to be explored (Figure 1).

### 4.4 Chemotoxicity of ICM and distinction from HSRs

Chemotoxic reactions arise from the physicochemical properties of ICM, such as high osmolality and viscosity, and can affect various organ systems, particularly the kidneys, with nephrotoxicity being of greater concern in patients with pre-existing renal impairment. Other potential chemotoxicity include cardiovascular changes (e.g., blood pressure or heart rate alterations) and gastrointestinal disturbances. The incidence of chemotoxic reactions has decreased with non-ionic, low-osmolality ICM, which are associated with fewer adverse events than high-osmolality ionic ICM (56). Unlike HSRs, chemotoxic reactions are not influenced by immune status or allergy history (57, 58), and recognizing the distinct mechanisms behind them is crucial for optimizing safety and improving diagnostic imaging outcomes (26, 31). Distinguishing between chemotoxic and anaphylactic HSRs during an acute reaction can be challenging, but it is possible with careful evaluation of dose-dependency, clinical presentation, and patient history (59, 60). Chemotoxic reactions are typically dose-dependent and present with mild, self-limiting symptoms, such as nausea, vomiting, warmth, or flushing. These reactions are distinct from anaphylactic HSRs, which are not dose-dependent and can occur even with minimal exposure, manifesting rapidly with symptoms like urticaria, angioedema, and bronchospasm, potentially progressing to anaphylactic shock (9, 61). Moreover, while HSR management typically involves premedication and switching to alternative ICM, preventing chemotoxicity focuses on minimizing the dose, adjusting the administration speed, ensuring adequate hydration, and using alternative imaging techniques when appropriate (35, 60, 62).

## 5 Clinical management of HSRs to ICM

### 5.1 Risk assessment and screening

#### 5.1.1 Patient characteristics

Several studies have shown that older age and female sex may be associated with a higher risk of HSRs to ICM, although this relationship may vary based on other factors and individual patient conditions. A multicenter study found a mean age of 59.1 years for those experiencing HSRs, with a higher prevalence in women (19). However, some studies suggested that male sex and age over 65 might offer protection against HSRs to ICM (15). Therefore, the impact of age and gender on the risk of HSRS to ICM needs further evaluation. A history of drug allergies or other allergic conditions is a well-established risk factor for HSRs to ICM. Individuals with a drug allergy history have an increased risk of HSRs to ICM, with an adjusted odds ratio (OR) of 3.5; notably, a previous ICM-related HSR strongly predicts future reactions, with an adjusted OR of 198.8 (19). Other significant risk factors for HSRs to ICM include respiratory allergies, chronic urticaria, and a history of adverse drug reactions (15). Moreover, genetic predisposition and comorbidities such as cardiovascular diseases, diabetes, and oncological status also increase the risk of HSRs to ICM (15, 30, 54, 55).

#### 5.1.2 The type of ICM and route of administration

The chemical structure and properties of ICM influence their potential to cause HSRs. For example, ICM such as iomeprol, iopromide, and iodixanol are more frequently associated with HSRs, with iomeprol showing a higher incidence of severe reactions compared to iopamidol and iohexol (15, 23, 63). Analysis of the FDA Adverse Event Reporting System Database revealed that iomeprol has the highest OR for HSRs, while iopromide and ioversol are more likely to cause angioedema, particularly in individuals aged 45 to 64 (26, 31, 64). The osmolality and ionic nature of ICM also contribute to the risk of HSRs. Non-ionic, low-osmolality ICM generally present a lower risk than ionic or high-osmolality counterparts, although variation exists (19, 24, 31, 65). For example, iohexol and ioversol exhibit higher HSR risks than iopamidol (26, 35). Meanwhile, the variability in the chemical structure of different ICM also impacts the type and severity of HSRs. Iodixanol is associated with a higher incidence of non-immediate reactions (12, 34), and iomeprol is associated with a higher incidence of severe cutaneous adverse reactions (29, 31, 65). Certain side chains, such as the N-(2,3-dihydroxypropyl) carbamoyl group, are linked to varying risks of HSR recurrence, and substituting ICM with different side chains has been shown to reduce the risk of severe reaction (24, 30, 65). Additionally, the route of ICM administration also affects HSR occurrence. A study involving 133,331 patients found that intravenous administration is associated with a higher frequency of HSRs than intra-arterial methods (66).

#### 5.1.3 Drug interactions

Drug interactions also contribute to HSRs to ICM, and many medications, such as beta-blockers, angiotensin-converting enzyme (ACE) inhibitors, and non-steroidal anti-inflammatory drugs (NSAIDs), have been discussed (15, 19, 30, 35). Beta-blockers and ACE inhibitors are essential in managing hypertension, heart failure, and other cardiovascular conditions, significantly reducing morbidity and mortality (67–70). Although concerns exist about their effects on anaphylaxis and allergic reactions (30, 35), their benefits typically outweigh the risks. Studies show these medications can be safely continued during ICM procedures without significantly increasing contrast-induced reactions (71, 72). Moreover, ACE inhibitors and angiotensin receptor blockers (ARBs) also provide nephroprotective effects by modulating the renin-angiotensin-aldosterone system (72). The decision to continue these medications should be individualized in clinical practice, as their benefits often outweigh the risks, even in high-risk patients (71, 73, 74). NSAIDs contribute by inhibiting cyclooxygenase enzymes, disrupting arachidonic acid metabolism, and promoting the production of leukotrienes, potent mediators of allergic reactions (30, 35). These medications may also compromise the body’s ability to respond to allergies, increasing the likelihood of severe outcomes (75). In contrast, corticosteroids and antihistamines effectively reduce HSR risk, especially in high-risk patients (35). Therefore, understanding medication histories and potential drug interactions is crucial for developing individualized premedication strategies or selecting alternative imaging ICM to minimize HSR risk. However, the exact doses of medications that increase or decrease HSR risk are currently unclear and require further exploration.

Overall, risk assessment and screening are vital in managing HSRs to ICM. By identifying risk factors through patient history and screening, clinicians can stratify patients based on risk levels and tailor preventive strategies (15, 19). Screening may involve diagnostic tools such as skin prick and intradermal tests, which help identify patients who may react to specific ICM and guide the selection of safer alternatives (39, 76). However, the sensitivity and specificity of these tests can vary and are more reliable for severe immediate reactions (32). Their negative predictive value is not always optimal, which may necessitate additional drug provocation tests in some cases (43, 48, 76). Additionally, a stratified assessment and warning regimen, which includes risk identification, stratification, early warning, and prevention, has been shown to reduce acute adverse reactions to ICM, enhancing patient safety during contrast-enhanced imaging (77, 78).

### 5.2 Premedication and alternative options

**Figure 2:**
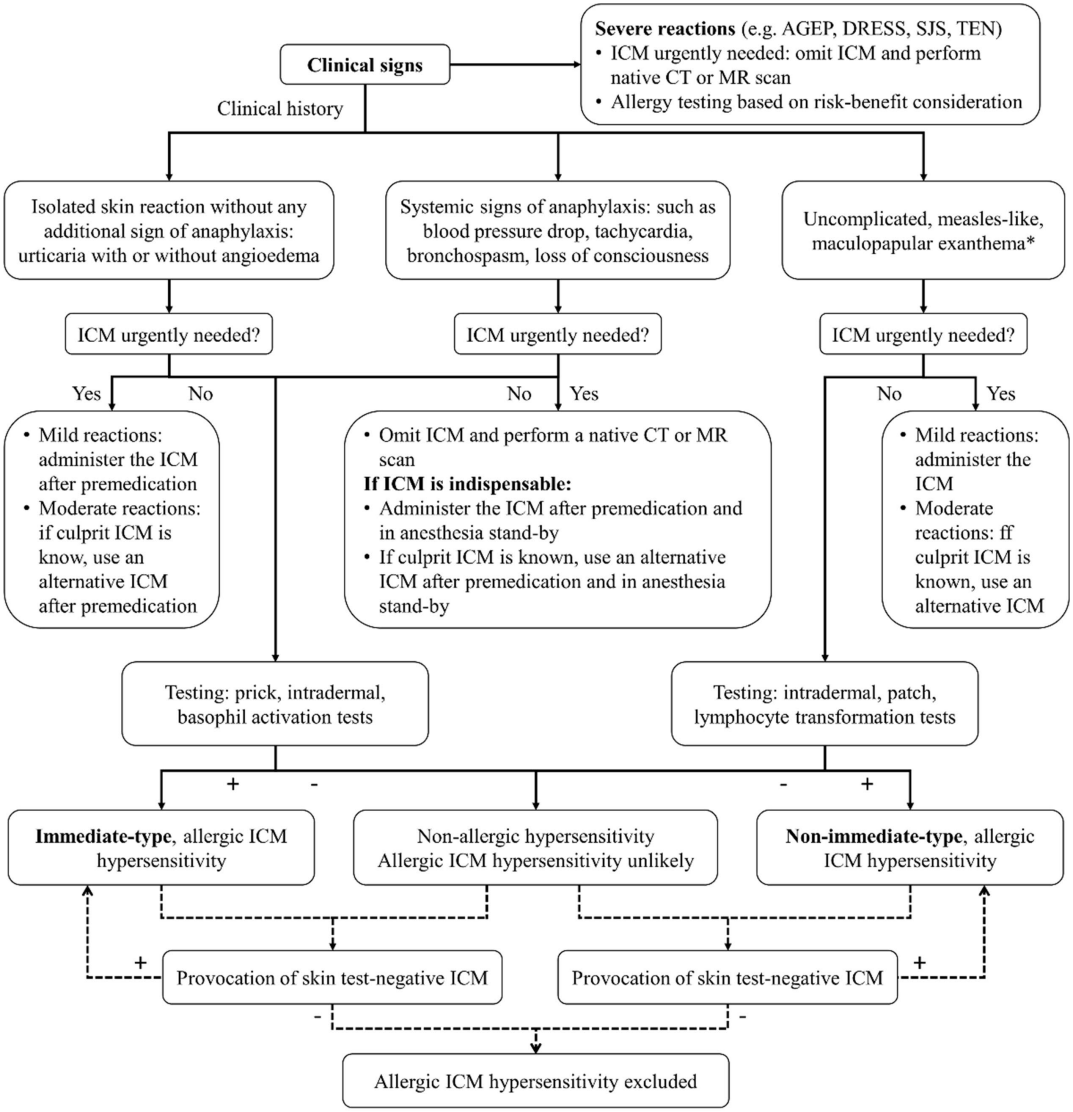
Algorithm for diagnosis and management of HSRs to ICM. ICM, iodinated contrast media; AGEP, acute generalized exanthematous pustulosis; DRESS, drug reaction with eosinophilia and systemic symptoms; SJS, Stevens-Johnson syndrome; TEN, toxic epidermal necrolysis; CT, computed tomography; MR, magnetic resonance. * or rarely: delayed urticaria and/or angioedema.

Preventive strategies for managing HSRs to ICM include premedication protocols and the selection of alternative ICM (Figure 2). The choice of premedication regimen can vary, and a common approach involves administering corticosteroids and antihistamines before ICM exposure, which has been shown to reduce HSR incidence, particularly in high-risk patients (35, 79, 80). However, the effectiveness of this regimen may vary depending on the severity of the initial reaction, and breakthrough reactions can still occur (35, 80–83). The recurrence of HSRs upon re-exposure to ICM is also a significant concern, and changing the type of ICM is a critical strategy, as specific ICM are associated with higher rates of HSRs (26, 31). A systematic review and meta-analysis found that switching from one ICM to another, such as from iohexol to iodixanol, was associated with a 61% reduction in the risk of recurrent immediate HSRs (29), suggesting that iodixanol may be a safer alternative for patients with a history of HSRs to iohexol. Opting for an ICM with a different chemical structure or side chain can further lower recurrence rates of severe HSRs (24, 29, 30), and this is especially beneficial for high-risk patients who have experienced moderate-to-severe reactions previously (30). In cases where patients have experienced previous HSRs to ICM, skin testing can be a valuable tool in identifying safe alternatives (9, 84). A combination of changing the culprit ICM and premedication has shown promising preventive outcomes (81), although cost and availability constraints may limit its widespread implementation (85).

### 5.3 Emergency management and treatment measures

Effective emergency management of HSRs to ICM requires prompt recognition and timely intervention to reduce morbidity and mortality. However, protocols for managing HSRs to ICM vary significantly across institutions due to differences in local practices, resources, and guideline adherence. A survey of Korean referral hospitals, compared with hospitals in other countries, highlighted these disparities. While most Korean hospitals appropriately performed informed consent and risk factor evaluation, there was notable variability in assessing renal function and using emergency equipment. For example, only 38.6% of Korean hospitals had a bronchodilator, compared to 100% of hospitals abroad. Moreover, 62.3% of Korean hospitals pre-medicated patients with a history of HSRs to ICM using antihistamines and corticosteroids, and 52.8% changed the culprit ICM (86).

The protocol variability can lead to inconsistent management, potentially increasing the risk of adverse outcomes. Standardizing management protocols across institutions is critical to ensuring patient safety and improving outcomes. The first step is to establish consensus-based clinical practice guidelines for diagnosing and managing HSRs to ICM (84, 87). By adopting standardized diagnostic and management protocols, institutions can ensure a consistent approach, minimizing variability in patient care and ensuring that all patients receive the best possible care regardless of where they are treated. Training healthcare professionals on these protocols, including symptom recognition, diagnostic tests, and emergency treatments, is essential. Regular audits and feedback can further support adherence and identify areas for improvement, fostering a culture of continuous learning to enhance preparedness.

For severe HSRs, which are rare but life-threatening, immediate steps include discontinuing the ICM and ensuring airway, breathing, and circulation. Intramuscular epinephrine is the first-line treatment and should be administered without delay, as it rapidly counters the severe allergic response by reducing airway swelling, increasing blood pressure, and improving heart function (35, 86). Supportive treatments such as intravenous fluids to stabilize blood pressure, antihistamines for urticaria and itching, and corticosteroids to mitigate the inflammatory response are also needed (17, 85). Although corticosteroids are commonly used in anaphylaxis due to their anti-inflammatory effects, there is limited evidence to support their routine use in preventing biphasic reactions, and their potential adverse effects complicate their use in this context (88). A study of Korean referral hospitals found that while most institutions were well-prepared, variability in bronchodilator availability highlighted the need for standardization in emergency protocols (86). Skin testing, which helps identify less reactive ICM for re-exposure, also plays a critical role in preventing severe HSRs (76).

### 5.4 Psychological support, patient education, and follow-up

HSRs to ICM can have significant psychological effects on patients, impacting their mental well-being and future healthcare interactions. The psychological impacts are often underestimated but can be profound, leading to anxiety, fear, and avoidance behaviors. Patients who have experienced an HSR to ICM may develop anxiety about future medical procedures requiring contrast media, driven by the fear of another life-threatening reaction. This anxiety can exacerbate stress levels and negatively affect their overall health. Studies have shown that patients with a history of HSRs are at a higher risk of recurrent reactions, further intensifying their anxiety (26, 29). The psychological effects can extend beyond the individual, impacting family members who may also develop concerns about the patient’s safety during medical procedures. This familial anxiety contributes to a heightened sense of vulnerability and stress, which can further affect the patient’s mental health (9, 17). Additionally, the psychological burden of HSRs can lead to avoidance behaviors, where patients delay or refuse necessary imaging due to fear of another reaction. This avoidance can delay the diagnosis and treatment of underlying health conditions, highlighting the importance of timely and accurate imaging.

Healthcare providers must be aware of these psychological effects and address them through patient education, reassurance, and preventive measures like premedication or alternative imaging strategies (8, 34). By incorporating psychological support into patient care, providers can mitigate the mental health impact of HSRs and improve the overall healthcare experience (24, 35). Patient education and follow-up are essential in managing HSRs to ICM. Educating patients about the risks of ICM and the importance of reporting any prior reactions allows healthcare providers to make informed decisions regarding contrast media use. A history of HSRs significantly increases the risk of future reactions, emphasizing the need for comprehensive assessments and patient education (17, 19). Follow-up care is critical for monitoring non-immediate HSRs, which may occur hours to days after exposure and are often underreported once the patient has left the medical facility. A structured follow-up protocol aids in the early identification and management of these delayed reactions, ultimately reducing the risk of severe outcomes (12, 34).

## 6 Controversies and future focus on HSRs to ICM

Current debates surrounding HSRs to ICM focus on prevention and management strategies. One major issue is the use of premedication, which is widely recommended to prevent HSRs. A systematic review and meta-analysis confirmed that corticosteroid premedication significantly reduces the recurrence of moderate to severe HSRs in high-risk patients (35). However, some studies suggest that premedication may be unnecessary in mild reactions (89). Some studies question the efficacy of premedication, particularly in patients receiving non-ionic ICM, where no significant difference in reaction rates between premedicated and non-premedicated groups was observed (90). Surveys revealed inconsistent premedication practices, especially in patients with severe allergies, underscoring the lack of consensus on its necessity (91). Moreover, breakthrough reactions still occur, indicating that premedication alone may not be entirely effective (85). Another point of contention is the choice of alternative ICM to reduce recurrent reactions. Evidence supports substituting the culprit ICM with one that has a different side chain to lower the risk of severe reactions (24), and substituting the culprit ICM, particularly with those lacking a carbamoyl side chain, can reduce recurrence by up to 69% (65). However, the optimal choice should be individualized based on the specific ICM involved (13). A multicenter study reported that combining ICM substitution with premedication is effective in reducing HSR recurrence (19).

Additionally, diagnostic challenges persist, particularly with skin tests like intradermal and patch tests, which, while specific, have limited sensitivity and can result in false negatives (92). Skin test-guided strategies involving intradermal tests to identify safe alternatives also show promise in reducing the frequency and severity of HSRs (13). Of course, comprehensive evaluations are needed, including clinical history and, when necessary, drug provocation tests, despite their associated risks (93). Therefore, while premedication remains common, its efficacy is debated, and alternative strategies such as ICM substitution or skin test-guided selection may offer additional benefits. Lastly, concerns regarding ICM as endocrine-disrupting chemicals have raised environmental and health issues, with the long-term effects on both human health and the environment still under-evaluated (51). Overall, these approaches emphasize the importance of personalized care in managing HSRs to ICM, and the choice of strategy should depend on individual patient risk factors and clinical judgment, highlighting the need for further research to refine these approaches and establish standardized guidelines.

Future research on HSRs to ICM should focus on several key areas. First, large-scale epidemiological studies are needed to better understand the prevalence, risk factors, and population-specific variations of HSRs, which could inform safer ICM use and clinical guidelines. Second, exploring the mechanisms underlying both immediate and non-immediate HSRs is essential. Understanding the cellular and molecular pathways could lead to targeted therapies and preventive strategies. Third, developing reliable predictive tools for identifying high-risk patients is crucial, including refining skin testing methods and investigating *in vitro* assays for more accurate risk prediction. Further research into genetic predispositions, drug interactions, and comorbidities will enhance risk stratification. Fourth, evaluating the effectiveness of current premedication protocols and exploring alternative strategies, such as different ICM or antihistamines, should be prioritized to improve prevention. Large-scale, multicenter allergic studies integrating skin and drug provocation tests will help identify safe alternative ICM, reducing unnecessary premedication risks and improving patient safety. Finally, the environmental impact of ICM, particularly their persistence in water systems and endocrine-disrupting potential, warrants further investigation. Research into advanced wastewater treatment technologies could mitigate these effects. Addressing these areas will improve the safety and clinical outcomes of ICM-based imaging procedures.

## 7 Conclusion

The incidence of HSRs to ICM varies significantly across studies, influenced by factors such as ICM type, patient demographics, study design, and geographical variations, highlighting the need for standardized methodologies to yield consistent and comparable prevalence estimates. HSRs to ICM can range from mild symptoms to severe, life-threatening complications with substantial clinical, psychological, and economic impacts. Mechanistically, HSRs to ICM are driven by both immune and non-immune pathways, with the chemical properties of ICM and patient-specific factors playing critical roles in their pathogenesis. Effective clinical management of HSRs to ICM involves a comprehensive and personalized approach, including risk assessment, preventive measures, emergency protocols, psychological support, patient education, and follow-up care. Controversies surrounding HSRs to ICM focus on the efficacy of premedication, the optimal choice of alternative ICM, and diagnostic challenges, with future research aimed at monitoring epidemiological trends, elucidating potential mechanisms, improving risk prediction, and refining and standardizing preventive, diagnostic, and therapeutic strategies to enhance patient safety and clinical outcomes. In conclusion, HSRs to ICM remain a complex and evolving clinical challenge, requiring a delicate balance between their indispensable role in diagnostic imaging and the imperative to mitigate associated risks.
